# 156. Evaluation of Trends in Antimicrobial Use and Proportion of Culture Positive Gram-Negative/Gram-Positive Pathogens Comparing Prior to and During the SARS-CoV-2 Pandemic: A Multicenter Evaluation

**DOI:** 10.1093/ofid/ofab466.358

**Published:** 2021-12-04

**Authors:** Laura A Puzniak, Karri A Bauer, Kalvin Yu, Vikas Gupta

**Affiliations:** 1 Merck & Co., Inc., Kenilworth, New Jersey; 2 Merck & Co, Inc, Kenilworth, New Jersey; 3 Becton, Dickinson and Company, Franklin Lakes, New Jersey

## Abstract

**Background:**

Increased risk for bacterial co-infections has been described in the pathogenesis of primary viral infections. We evaluated trends in incidence of antibiotic use (abx) and culture positive Gram negative/Gram positive (GN/GP) pathogens in US hospitalized patients prior to and quarterly during the SARS-CoV-2 pandemic.

Table. Trends in antimicrobial use, duration, and positive GN/GP pathogen results.

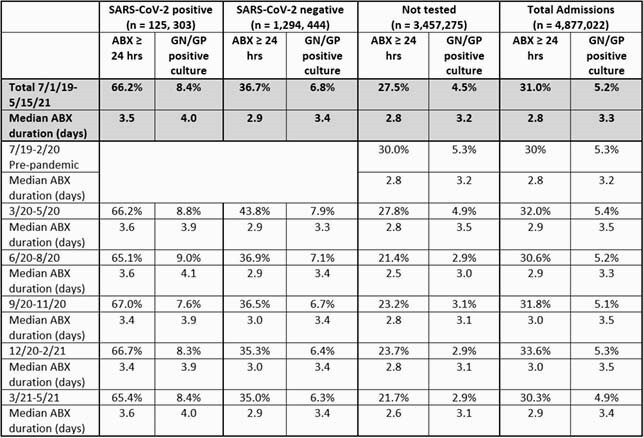

**Methods:**

We conducted a multi-center, retrospective cohort analysis of all hospitalized patients from 241 US acute care facilities with >1-day inpatient admission between 7/1/19-5/15/21 in the BD Insights Research Database (Franklin Lakes, NJ USA). SARS-CoV-2 infection was defined as a positive PCR during or ≤7 days prior to hospitalization. Admissions with abx prescribed ≥24 hrs and a GN/GP non-contaminant, positive culture were evaluated.

**Results:**

During the pre-pandemic period (7/19 – 2/20) 30% (600,116/2,001,793) admissions were prescribed abx ≥ 24 hrs and 5.3% were positive for a GN/GP pathogen (Table 1). During the SARS-CoV-2 pandemic, abx use ≥ 24 hrs (66.2%) and positive GN/GP culture (8.4%) was highest in SARS-CoV-2 positive patients followed by patients negative for SARS-CoV-2 (abx ≥ 24 hrs 36.7%; GN/GP pathogens 6.8%), and SARS-CoV-2 not tested (abx ≥ 24 hrs 27.5%; GN/GP pathogens 4.5%). GN/GP positive culture was consistent by quarter during the pandemic for SARS-CoV-2 positive patients, whereas SARS-CoV-2 negative and not tested patients had the highest proportion of antibiotics received and positive pathogens in the first three months of pandemic. SARS-CoV-2 positive patients with positive GN/GP culture had the longest median abx duration. (Table 1) The prevalence of abx usage was highest in all groups for all abx during the early pandemic and then declined over time with the largest declines in SARS-CoV-2 positive patients. (Table 2)

**Conclusion:**

This study highlights the impact of viral infections on both prescribing practices and prevalence of bacterial pathogens. Approximately two-thirds of SARS-CoV-2 positive patients received an antibiotic despite a low percentage of positive cultures, however aggregate antimicrobial use overall was similar prior to compared to during the SARS-CoV-2 pandemic. These data may inform opportunities for stewardship programs and antibiotic prescribing in the current and future viral pandemics.

**Disclosures:**

**Laura A. Puzniak, PhD**, **Merck & Co., Inc.** (Employee) **Karri A. Bauer, PharmD**, **Merck & Co., Inc.** (Employee, Shareholder) **Kalvin Yu, MD**, **BD** (Employee) **Vikas Gupta, PharmD, BCPS**, **Becton, Dickinson and Company** (Employee, Shareholder)

